# An mHealth Intervention Using a Smartphone App to Increase Walking Behavior in Young Adults: A Pilot Study

**DOI:** 10.2196/mhealth.5227

**Published:** 2016-09-22

**Authors:** Jane C Walsh, Teresa Corbett, Michael Hogan, Jim Duggan, Abra McNamara

**Affiliations:** ^1^ mHealth Research Group School of Psychology National University of Ireland, Galway Galway Ireland; ^2^ National University of Ireland, Galway Galway Ireland

**Keywords:** mHealth, physical activity, mobile phone app, intervention, health behavior change, feasibility study

## Abstract

**Background:**

Physical inactivity is a growing concern for society and is a risk factor for cardiovascular disease, obesity, and other chronic diseases.

**Objective:**

This study aimed to determine the efficacy of the Accupedo-Pro Pedometer mobile phone app intervention, with the goal of increasing daily step counts in young adults.

**Methods:**

Mobile phone users (n=58) between 17-26 years of age were randomized to one of two conditions (experimental and control). Both groups downloaded an app that recorded their daily step counts. Baseline data were recorded and followed-up at 5 weeks. Both groups were given a daily walking goal of 30 minutes, but the experimental group participants were told the equivalent goal in steps taken, via feedback from the app. The primary outcome was daily step count between baseline and follow-up.

**Results:**

A significant time x group interaction effect was observed for daily step counts (*P*=.04). Both the experimental (*P*<.001) and control group (*P*=.03) demonstrated a significant increase in daily step counts, with the experimental group walking an additional 2000 steps per day.

**Conclusions:**

The results of this study demonstrate that a mobile phone app can significantly increase physical activity in a young adult sample by setting specific goals, using self-monitoring, and feedback.

## Introduction

### Physical Activity Levels in Young Adults

Physical activity (PA) is an important factor in maintaining the health and wellbeing of the population [1]. Physical inactivity is associated with over 20 chronic diseases, including coronary heart disease, diabetes, and cancer. PA patterns established in childhood tend to be maintained into adulthood [2]. Recent research suggests that over 40% of students are physically inactive [3] and only 13-32% of this group meet the recommended PA guidelines [1,2]. The percentage of students classified as overweight or obese in the United States increased from 29% to 32.5% between 2000 and 2009, and this trend is set to continue [4]. Young people demonstrating low levels of PA are significantly more likely to be overweight after university, and may experience negative side effects, including the influence of body shape on both income or occupational attainment [4]. Overweight or obese individuals may also experience many negative emotional and social consequences, including depression, stigmatization, and lower academic achievement [5]. The promotion of increased PA should be a priority for university students. Not only does PA play an important role in maintaining physical, psychological, and social wellbeing [2], it can also enhance cognitive performance in students [6].

### Pedometers and Physical Activity

Previous research has suggested that the daily step count for healthy adults ranges from 6000 to 7000 steps per day [7], although this falls short of recommended guidelines of 10,000 steps per day [8]. Walking is often encouraged as a simple solution to physical inactivity [9]. PA interventions are more likely to cost-effective when they are easy to perform, and walking has been rated as the most favored form of PA by sedentary groups [10]. Walking is a low impact activity in which the individual can control intensity (low, moderate) or exertion to reduce the risk of injury, and can be easily incorporated into most daily routines with minimal risk [11]. Pedometers (or devices to count steps) also offer a way to monitor steps, and thus provide direct feedback on daily walking patterns. Pedometers (via mobile devices) are increasingly used, and the results of recent meta-analyses suggest that this technology is an excellent means to increase PA [12,13] with interventions delivered via mobile phones, yielding significant moderate effects (*g=*.52, 95% CI 0.11-0.94,  *P*=.01) [13].

### mHealth Interventions and Physical Activity

Mobile health (mHealth) involves public health initiatives that are supported by mobile devices (eg, mobile phones, tablet computers), and is increasingly being used as part of public health interventions, including PA interventions [14]. Novel mHealth technologies (eg, FitBits, Garmin watches, mobile phones), and in particular mobile phone apps, offer new possibilities that facilitate users to engage in behaviors such as planning, goal-setting, self-monitoring, and receiving continuous feedback (eg, step counts, calories burned). A recent study reported that 96% of Irish 15-35 year-olds owned a smartphone [15], thus providing significant opportunities for mHealth initiatives. Mobile phones with pedometer-enabled apps are useful, as people usually carry these devices throughout the day. Mobile apps also move beyond traditional forms of pedometer monitoring, with additional features such as automatic feedback, tracking of steps and calories, appealing graphic displays, and goal-oriented functionality [16].

Bort-Roig et al [17] highlighted the potential for PA interventions using smartphones. Glynn and colleagues [14] conducted the SMARTMOVE trial, one of the first studies to use smartphone technology to promote PA in patients with chronic disease. This study found that an app used in a primary care setting increased PA, decreased weight, and decreased blood pressure compared to controls [14]. The results from a qualitative study conducted with trial participants suggested that use of the app facilitated an interactive process of positive change in participants (and their exercise behavior) via the goal-setting and feedback dimensions of the app [18]. The authors called this the *Know-Check-Move* effect, and described how an app can affect behavior change through an increase in awareness and knowledge, goal-setting, and the use of feedback. A recent meta-analysis that focused on increasing PA with mobile devices found that the use of mobile technology was an effective means of influencing PA behavior [13]. However, the authors suggested that interventions should focus on selecting the best possible use of these tools to measure and understand behavior. Therefore, theoretically grounded behavior change interventions that recognize and act on the potential of mobile phone technology could provide investigators with an effective tool for increasing PA [13].

### Using a Theory-Based Approach in Health Behavior Change

The aforementioned findings agree with the *Behavior Change Taxonomy*, which identified key behavior change techniques (BCTs) for health behavior change interventions [19]. BCTs including education, goal-setting, and modelling, are observable and replicable components of behavior change interventions. While mHealth interventions hold significant potential, adopting a theory and evidence-based approach to intervention design is critical.

A recent review found that the use of relevant BCTs significantly increased the success of weight loss programs [20]. Students are among the most frequent users of smartphones and present a major opportunity to promote healthy lifestyles [21]. Although research evaluating mHealth interventions designed to increase PA is in its infancy, findings to date are promising.

This study sought to extend the findings of Glynn et al [14] by examining the feasibility of this approach by using the *Accupedo-Pro Pedometer* app intervention to promote PA in a healthy student sample. It was hypothesized that encouraging participants to engage with particular features of an app (eg, BCTs of goal-setting and self-monitoring) would significantly increase step counts compared to giving standard PA recommendations.

## Methods

### Design

This study used a *time x group* mixed design (baseline and 5-week follow-up; control and experimental groups). The dependent variable was mean daily step count. Ethical approval for the study was granted by the National University of Ireland Galway School of Psychology, Research Ethics Committee.

### Sample Size and Recruitment

**Figure 1:**
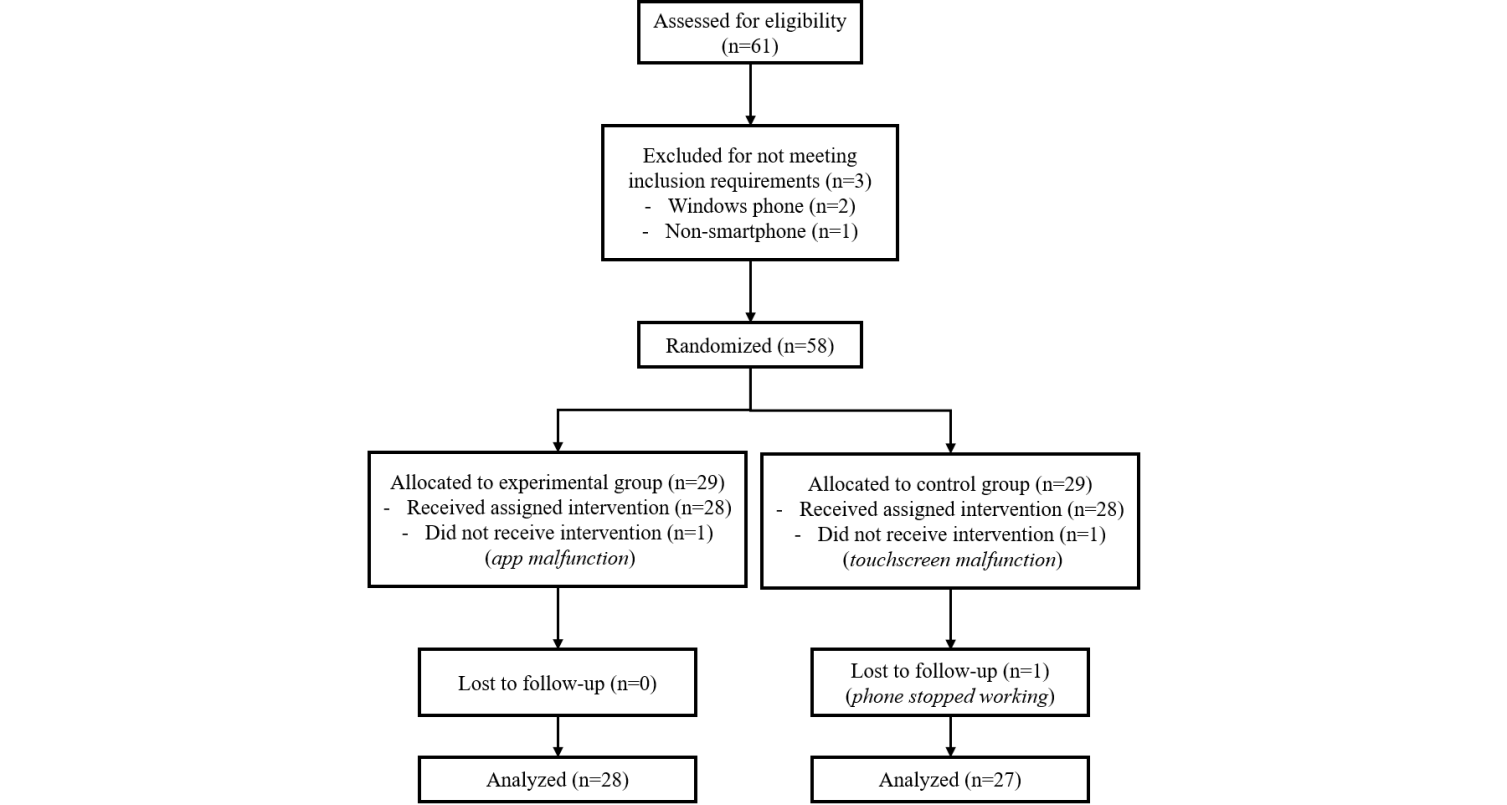
Participant flow from recruitment to follow-up and analysis.

In order to reach a 95% confidence level in a pilot study, the estimated sample size was 59 young adults, who were randomized to receive either the intervention or usual care [22]. A final sample of 61 young adults was recruited through the university’s research participation website. Students were given course credits for participating in the study. Participants were eligible for the study if they owned a smartphone (iPhone or Android). Three participants were initially excluded due to owning a non-compatible mobile phone, and two were lost before follow-up due to their phone malfunctioning. Information regarding participants’ flow from recruitment to follow-up is displayed in Figure 1.

### Measures

**Figure 2:**
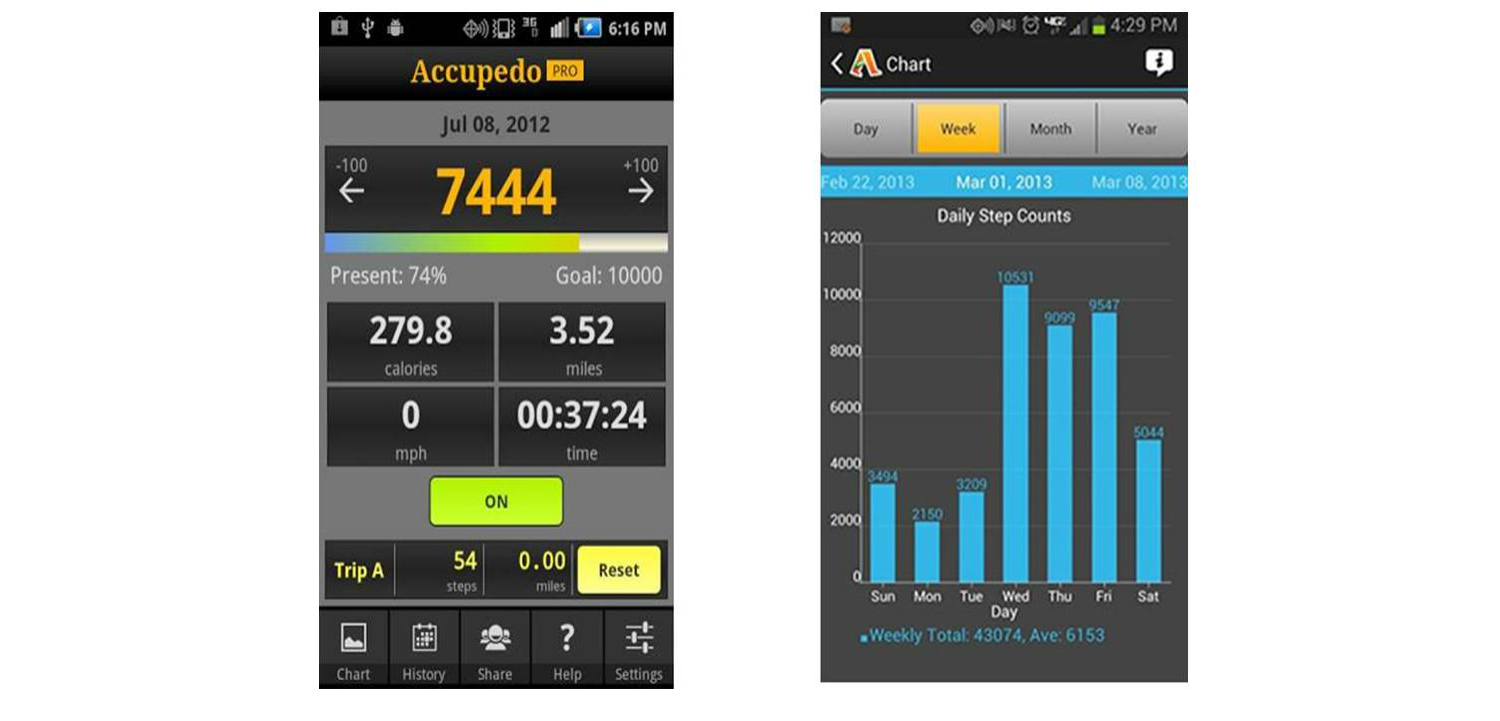
Accupedo Pedometer app screen shots.

Daily step-counts were measured using *Accupedo-Pro Pedometer* app (see Figure 2); this app can give feedback on distance, time, speed, and calories burned. *Accupedo* can also be operated to run in the background of the mobile phone without the display being visible.

### Mobile Phone App and Selection Process

The *Accupedo-Pro Pedometer* app was selected for use in the current study. Notably, this app obtained the highest ratings in previous comparisons of pedometer apps [16]. This result was based on key criteria for promoting PA, including: automatic feedback and tracking of step counts and calories burnt; visually appealing graphic display of step-count history; goal-setting functionality; and goal-achievement feedback [16].

### Demographics

The gender and age of participants were recorded using a demographic questionnaire that was distributed at the first meeting.

### Procedure

#### Intervention Development

The Capability, Opportunity, Motivation, Behavior (COM-B) framework is a simple model that hypothesizes that an interaction between three components (capability, opportunity, and motivation) influence behavior, and can provide explanations for why a recommended behavior is not undertaken [23]. The COM-B model and the Behavior Change Wheel [23] were used as guides during the intervention design process, and aided in the identification of BCTs. The Behavior Change Wheel identifies intervention functions that target relevant components of the COM-B model. In this trial, education, training, and modelling were used to address the psychological capabilities and reflective motivation of the students, in order to increase walking behavior. The complete methodology of the study can be found in Multimedia Appendix 1.

#### Baseline Assessment

Details of the procedure are described in Figure 1. Participants were self-selected via the university research website, or contacted the researcher to make an appointment. A randomization code was assigned to each participant, and relevant demographics and other data were collected. Participants were then assigned to either the control or experimental group via block randomization (which was used to guarantee similar numbers within each condition).

All participants then had the app downloaded onto their mobile phones to record their daily step counts, in order to provide a measurement of their baseline PA levels. For the week following the screening visit (*Week 1*), all participants were asked to carry their mobile phone during waking hours and to continue operating at their normal PA levels. During *Week 1*, the mobile phone app display was not visible to either group and the investigators remained blinded. At the end of this period each participant met with a researcher, who used the *share* option on the app to receive the previous week’s step-count data.

Following the collection of baseline data, the randomization code was broken by the investigators. Both intervention and control groups were then given similar PA goals and information related to the benefits of exercise; however, only the intervention group was told how to use the app to help them achieve these goals.

#### Control Group

Participants in the control group were provided with information related to daily recommended PA levels (ie, 30 minutes daily), and information highlighting the benefits of walking regularly [8]. The control group was given a goal of 30 minutes of walking per day over the following month. The control group continued to have the pedometer app running in the background of their phone so that it continued to record their daily steps, without being visible or requiring interaction.

#### Intervention Group

Participants in the intervention group were provided with the same information as the control group regarding the benefits of walking and daily recommended PA levels. This group was encouraged to achieve a target goal of 10,000 steps per day, and were informed that this value was roughly equivalent to 30 minutes of walking per day (along with their normal activity). Researchers also demonstrated the usability features of the mobile phone app to the intervention group (using standardized instructions), and encouraged this group to use the app to monitor their steps and obtain feedback, in order to achieve their target goals. The follow-up meetings for both groups took place five weeks after the baseline data was taken. Data was collected in full, and participants were fully debriefed.

### Statistical Analyses

A *time x group* (baseline and 5-week follow-up; control and experimental groups) mixed-analysis of variance (ANOVA) was conducted to analyze group differences in step counts over time. Post hoc *t*-tests were conducted to compute group differences. Analysis of missing data suggested that information was missing at random, and was therefore accounted for with Expectation Maximization substitution in the mixed model; the validity of this assumption was investigated by examining the missing data patterns and by modelling the probability of missing data based on the explanatory variables available. Tests of normality demonstrated that the data was normally distributed at both time points for step counts (Shapiro-Wilk *P*=.07 for both groups). Tests for equality of variance also indicated that this assumption was met (Levene’s test *P*=.16).

## Results

### Baseline Characteristics

The final sample included a total of 55 participants (40 female, 15 male) between the ages of 17 and 26 (mean 20.55, standard deviation [SD] 2.07). Attrition was low (n=3), and was solely attributed to app or mobile phone malfunctions. Details regarding participant dropouts are displayed in Figure 2. Descriptive statistics for each condition can be found in Table 1.

Results of an independent samples *t*-test showed that there was no significant difference between daily step counts of the control and experimental groups at baseline (*t*_53_=.85, *P*=.401), ensuring that randomization was effective.

**Table 1:** Descriptive statistics of demographics.

	Age	Gender	
	Years, Mean (SD)	Male, n (%)	Female, n (%)
Control group (n=27)	20.30 (1.73)	7 (25.9)	20 (74.1)
Experimental group (n=28)	20.79 (2.36)	8 (28.6)	20 (71.4)
Total (N=55)	20.50 (2.07)	15 (27.3)	40 (72.7)

### Changes in Physical Activity (Step Counts)

**Figure 3:**
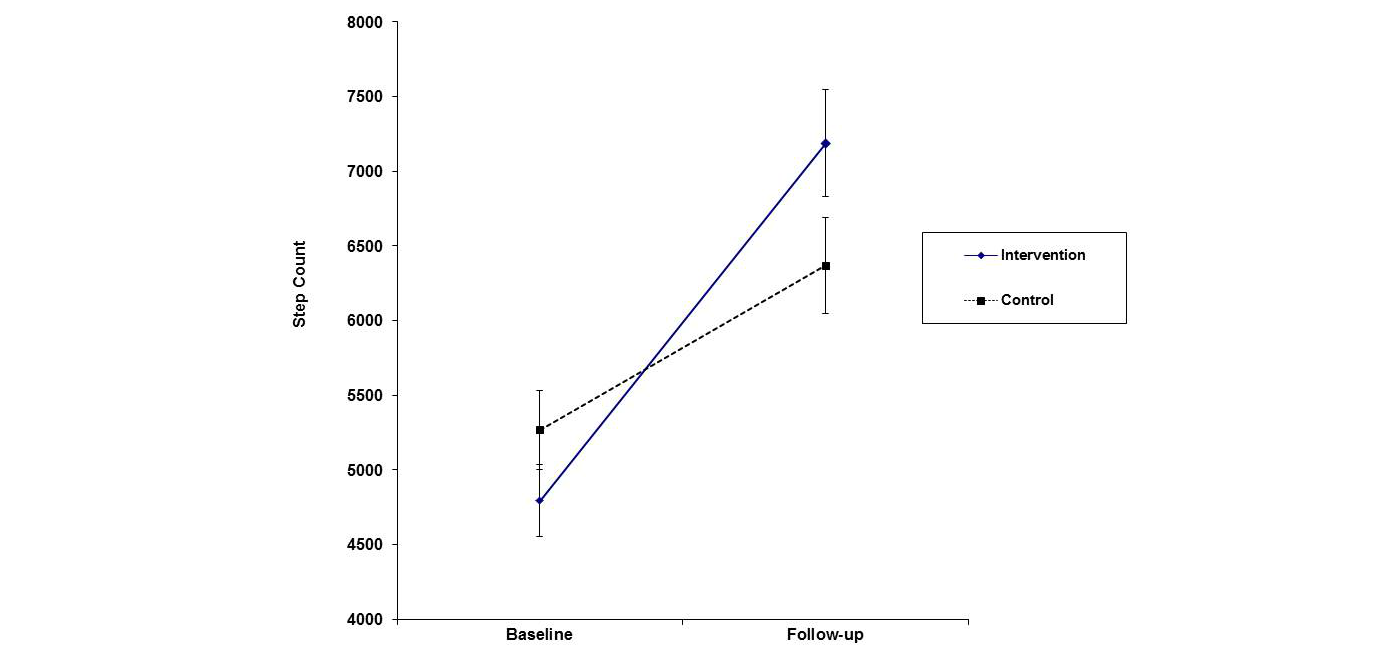
Graph showing interaction effect between time and condition.

A *time x group* mixed ANOVA was conducted to examine the impact of the experimental condition on daily step-counts from baseline to 5-week follow-up. A significant interaction effect was found between time and condition (*F*_1,53_=4.30, *P*=.043, η_p_^2^=.08). A between-group *t*-test of the differences in step counts from baseline to follow-up revealed that participants in the intervention condition had a significantly higher increase in step count (2393) than those in the control condition (1101; *t*_53_=2.07, *P*=.043; see Figure 3). Dependent samples *t*-tests revealed a significant increase in daily step counts from baseline to follow-up for both the control (*t*_26_= -2.25, *P*=.033) and the experimental group (*t*_27_= -6.14, *P*<.001). A main effect was found for time (*F*_1,53_*=* 31.43, *P*<.001, η_p_^2^=.37), with both the control and experimental groups achieving a higher daily step count at follow-up (mean 6785.55, SD 2815.37 *)* compared to baseline (mean 5026.78, SD 2071.86). No main effect was observed for in overall group differences (*F*_1,53_= .09, *P*=.77).

## Discussion

Findings from this pilot study indicate that an intervention using a mobile phone pedometer app resulted in a significant increase in PA (step counts) in young adults over and above the provision of basic information on PA recommendations. The intervention group achieved a significant increase of over 2300 steps per day (an increase of approximately 45% in activity levels), equivalent to a distance greater than one mile. The medium effect size and increase in step counts were comparable to results found in a recent meta-analysis, which found that the use of pedometers had a moderate effect size (0.68, 95% CI 0.55-0.81) on the increase of PA in intervention studies [12]. This substantial change in behavior, if maintained, could result in numerous health benefits, including a reduced risk for obesity and cardiovascular disease [24]. Both the control and experimental groups displayed a significant increase in step counts during the study period. This effect was significantly greater for participants in the intervention group who had a target goal of 10,000 steps per day, and interacted with the self-monitoring aspects of the app. Findings suggest that pedometer-based interventions targeting college-aged cohorts may facilitate increases in their levels of PA over a relatively short period of time (one month).

Notably, baseline levels of activity were low in both study groups (approximately 5000 steps/day), which was less than the daily step count previously shown for healthy adults (between 6000 and 7000 steps/day) [8]. By the end of the intervention, both groups had reached the minimum guideline targets, suggesting that pedometer apps are an effective mode of PA promotion for young adults at a life-stage when it is important to develop healthy behaviors.

### Relationship with Other Research

Recent studies have highlighted the role of walking as it relates to increasing PA at a population level [10]. Heron et al [9] highlight the potential for pedometers as one technology to facilitate this activity. In particular, for students who frequently engage with apps and are generally healthy, these types of interventions may be critical in promoting activity in non-healthcare settings.

The steepest decline in PA occurs during adolescence and early adulthood [25]. World Health Organization (WHO) guidelines propose that inactive adults will have added health benefits with only minor behavioral changes, such as a shift from *no activity* to *some levels of activity*. Young adults who do not currently meet the recommendations for PA are encouraged to increase duration and frequency to achieving these goals [1]. Promotion of healthy behaviors in young adults is a priority in line with the WHO global strategy to protect health through PA, in order to substantially reduce disease burden in later years [1]. These changes may be facilitated by pedometer apps.

In this study, the control group had a significant increase in step-counts that was sustained over time. Simply using the app or participating in the study may have been sufficient to increase step-counts or impact motivation in both groups. Brunet and Sabiston [26] suggest that PA interventions may benefit from promoting or maintaining autonomous regulations within young-adult populations. Glynn et al [14] found that the control group in their study showed an initial increase in step count, which then decreased back to baseline. A longer follow-up period may be required to examine how students’ step-counts might change over time.

This study confirms that a significant increase in steps per day is achievable in a relatively inactive healthy sample [7]. This study is one of the first to consider the importance of mHealth technology tools to promote PA as a preventative measure in a healthy sample. The medium effect size and increase in step counts were equivalent to the results found in a recent meta-analysis, which found that the use of pedometers had a moderate effect size on the increase of PA in intervention studies [12].

### Strengths and Limitations of the Study

One of the key strengths of this study is the use of taxonomy to describe the intervention content, and the selection of established BCTs that are associated with positive effects in the literature relating to the promotion of PA. Additional strengths of this study include the randomized design, limited exclusion criteria, and low attrition rates. Participants were only excluded due to technological issues (ie, incompatibility of mobile phone with the app or phone malfunction), therefore strengthening external validity.

This study also had a number of limitations, including (1) self-selection of the sample, (2) limited generalizability of results (given the focus on college students), (3) short follow-up timeline, and (4) use of an active control group. A longer follow-up period would have provided clearer insights into the maintenance of the behavior changes, however the 5-week follow-up in this study was comparable to that of Glynn et al [14]. The use of an active control group was deemed to be appropriate for a pilot study, as participants in the control group were provided with freely available information relating to the benefits of walking and daily recommended PA levels (eg, government guidelines on recommended levels of PA). Ideally a passive control group would also be included in a full trial.

The 10,000 steps/day target has been criticized as a universal step-goal due to differences across different subgroups [8]. Wilde et al [27] argue that walking-based interventions should incorporate personalized step-goals. Given that the participants in this study were quite sedentary at baseline, it may have been more beneficial to provide them with easily achievable personalized goals that could be readjusted over time [28], rather than setting the same 10,000 step-goal for every participant. Recent findings also suggest that focusing on reducing sedentary behaviors may lead to a greater reduction in sedentary time, compared to interventions that focus on increasing PA [29].

### Future Research

In future adaptations of this study, the target aims of the study and individual goals should be tailored to the participants’ baseline level of activity, and incorporate psychosocial factors (eg, motivation or self-efficacy), in order to increase walking behavior [30]. It may be of interest to compare traditional wrist pedometers to an app, or to include objective measures of fitness, such as maximal oxygen uptake or heart rate.

### Conclusions

This study has provided information on the use of a pedometer app in a community setting to promote PA, and found that students were willing to engage with the app to promote PA. This finding is encouraging for a larger research study or trial. It is important to encourage PA in inactive young adults, as PA levels [31,32] and PA motivation and enjoyment [33,34] may decrease gradually with age. The use of a pedometer app appeared to increase the number of steps taken per day by this relatively inactive group, even over a short period of time.

Mobile phone apps offer the potential to reach a large population through accessible, user-friendly, and autonomous self-monitoring means. A greater understanding of how these tools may be harnessed to promote positive behavior change is required. It is necessary to identify the most effective means of promoting PA for individuals that use their apps most frequently, before focusing on groups that are more difficult to engage.

## Appendix

Multimedia Appendix 1 Information on methodology.

## Abbreviations

ANOVA: analysis of variance
BCT: behavior change techniques
COM-B: Capability, Opportunity, Motivation, Behavior
mHealth: mobile health
PA: physical activity
SD: standard deviation
WHO: World Health Organization
