# Supplementary material for: An mHealth Intervention Using a Smartphone App to Increase Walking Behavior in Young Adults: A Pilot Study
Source: JMIR Mhealth Uhealth. 2016 Sep 22;4(3):e109. doi: 10.2196/mhealth.5227 (PMC5054234; doi:10.2196/mhealth.5227)
Supplement: Multimedia Appendix 1 [file mhealth_v4i3e109_app1.pdf]

Multimedia Appendix 1. Information on methodology.

Ethical approval obtained.		
Participants recruited		
Screened by the research team for inclusion suitability.	Inclusion criteria:<br>Students in the university<br><br>Active smartphone users<br>>16 years of age	Exclusion criteria:<br>Acute psychiatric illness / unable to undertake moderate exercise<br>Pregnancy<br>Not an owner of a suitable smartphone
Baseline screening meeting:	Participants given study information, signed consent, and smartphone application (app), Accupedo-Pro Pedometer, was downloaded to smartphones step-count display not made visible.	
Week 1:	All participants continued normal activity level; carried smartphone during all waking hours so the app could record baseline step count while remaining invisible to the participant.	
End of week 1:	Randomization code broken; participants assigned to control or intervention groups.	
	Intervention group:<br>App and step count made visible<br>Given information on benefits of exercise<br>Instructed to increase physical activity (goal of 10 000 steps per day); Encouraged to use app to achieve goal	*Control group*:<br>App with step count continues to remain invisible<br>Given information on benefits of exercise<br>Instructed to increase physical activity with a goal of an additional 30 minutes' walking exercise (equivalent to 10 000 steps) per day
BCTs used in each study arm	1.3 Goal setting (outcome)<br>1.6 discrepancy between current behavior and goal<br>2.2 feedback on behavior<br>2.3 Self-monitoring of behavior<br>4.1 Instruction on how to perform a behavior<br>5.1 Information about health consequences<br>6.1 Demonstration of the behavior<br>5.1 Information about health consequences	1.3 Goal setting (outcome)<br>2.3 Self-monitoring of behavior<br>4.1 Instruction on how to perform a behavior<br>5.1 Information about health consequences<br>6.1 Demonstration of the behavior

COM-B model targets of intervention	Psychological capability	Psychological capability<br>Reflective Motivation
Intervention Functions identified using the Theoretical Domains Framework	Education	Education<br>Training<br>Modelling
After completion of 5-week trial:<br>Quantitative results analyzed with SPSS for outcome	Stepcount for all participants recorded; After trial completion, all control-group participants shown how to use the app.<br>mean difference in daily step count between baseline and 8-week follow up	

SPSS = Statistical Package for the Social Sciences.
